# Supplementary figures and images for: High frequency electrical stimulation induces a long-lasting enhancement of event-related potentials but does not change the perception elicited by intra-epidermal electrical stimuli delivered to the area of increased mechanical pinprick sensitivity
Source: PLoS One. 2018 Sep 6;13(9):e0203365. doi: 10.1371/journal.pone.0203365 (PMC6126845; doi:10.1371/journal.pone.0203365)

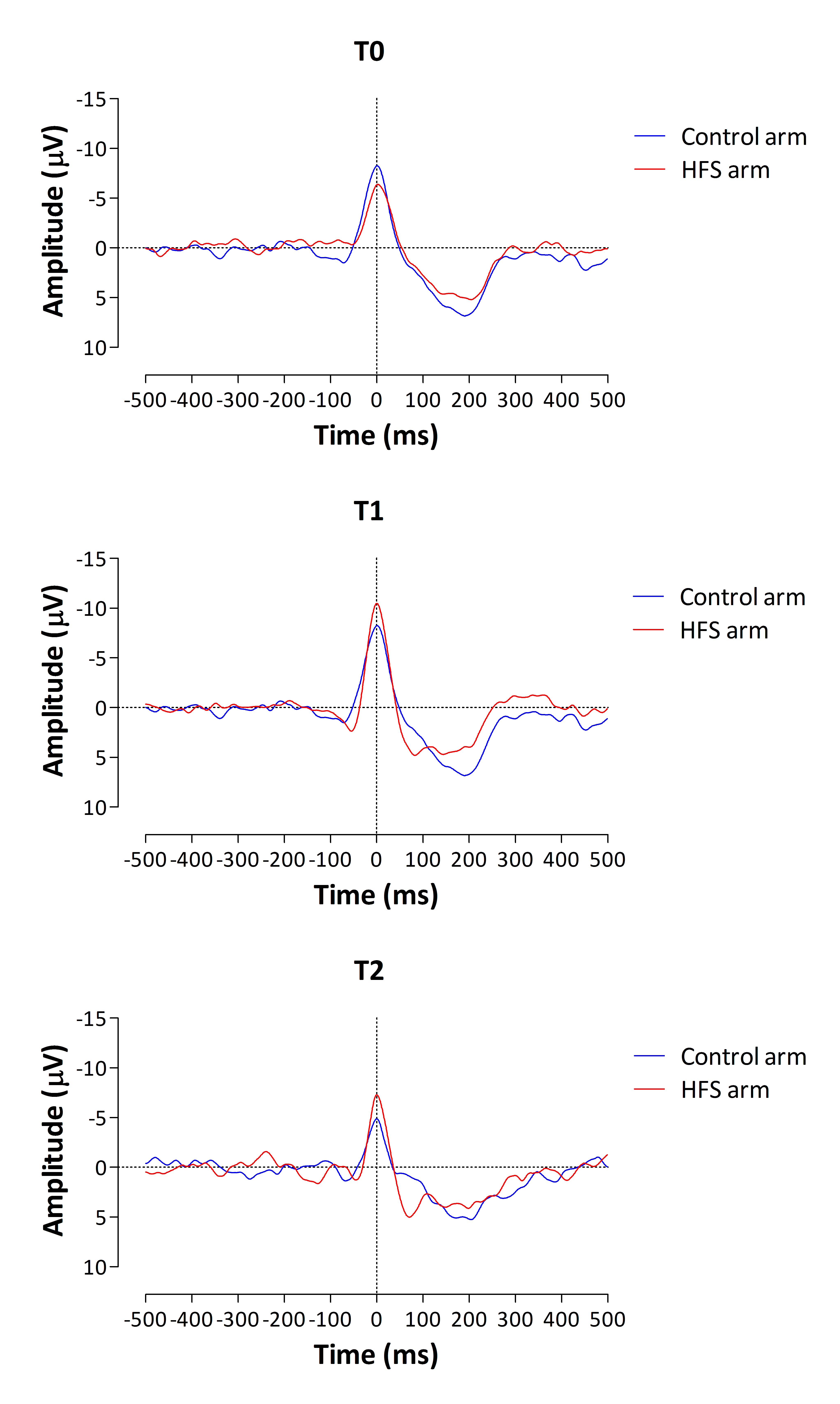

Supplement: S1 Fig — Because of the large inter-individual variability in the latency of the ERP responses we aligned, for each condition separately, all the individual ERPs to their own N2 peak latency. (TIF) [file pone.0203365.s001.tif]
